# The Role of Endogenous Antimicrobial Peptides in Modulating Innate Immunity of the Ocular Surface in Dry Eye Diseases

**DOI:** 10.3390/ijms22020721

**Published:** 2021-01-13

**Authors:** Youssof Eshac, Rachel L. Redfern, Vinay Kumar Aakalu

**Affiliations:** 1 Faculty of Medicine, Alexandria University, Alexandria 21131, Egypt; yeshac@outlook.com; 2 The Ocular Surface Institute, College of Optometry, University of Houston, Houston, TX 77204, USA; rredfer2@central.uh.edu; 3 Department of Ophthalmology and Visual Sciences, College of Medicine, University of Illinois at Chicago, Chicago, IL 60612, USA

**Keywords:** ocular surface disease, antimicrobial peptide, innate immune system, dry eye disease

## Abstract

The ocular surface has the challenging responsibility of maintaining a clear moist refractive surface while protecting the eye from exogenous pathogens and the environment. Homeostasis of the ocular surface, including its innate immune components, is altered in ocular surface disease states. In this review, we focus on antimicrobial peptides and the role they play in the immune response of the ocular surface during healthy states and dry eye diseases. Antimicrobial peptides are of special interest to the study of the ocular surface because of their various roles that include microbial threat neutralization, wound healing, and immune modulation. This review explores current literature on antimicrobial peptides in ocular surface diseases and discusses their therapeutic potential in ocular surface diseases and dry eye.

## 1. Introduction

The ocular surface is a complex and delicately balanced system which allows the eye to interact with and remain protected from the environment. Changes in this system are seen in many ocular surface diseases (OSDs). Emerging information suggests that antimicrobial peptides (AMPs) could play a role in the ocular surface inflammatory response in OSDs, like dry eye disease (DED). In DED, ocular surface homeostasis is disrupted and hyperreactivity to environmental triggers may worsen inflammation DED [1]. The hallmark of DED is the presence of tear film hyperosmolarity which can stimulate an inflammation to further disrupt the tear film. If inflammation is left unchecked, the disease can then enter a self-propagating “vicious cycle” leading to chronic ocular surface damage and inflammation. At the heart of the inflammatory response are the mechanisms that stimulate the production of inflammatory mediators and cytokines [2]. The production of AMP is variably altered in the diseased state, and the study of these patterns is essential to the understanding of the pathogenesis of DED and possible novel therapeutic avenues. In this review, we describe the roles of AMPs in normal and disease states of the ocular surface. In particular, we focus on the interplay between AMPs, innate immune responses, and environmental pathogens and how these elements can inform our understanding of OSDs.

Providing a clear, moist refractive surface that is exposed to the outside world while preventing infection and tolerating environmental elements is a challenging role for the ocular surface [3]. This role is made more important because of the need for a healthy epithelium for clear vision. One of the means by which the ocular surface protects itself from the environment is through its innate immune system. The innate immune system consists of physical and chemical barriers as the first line of defense. Anatomically, the eye is protected by eye lashes, eyelids, tear film, and the ocular surface epithelium. The tear film barrier of the ocular surface has the responsibility of lubricating the ocular surface, and among other elements, it contains important AMPs, secretory antibodies, and complement. The ocular surface also possesses a number of factors, such as lysozyme, lactoferrin, and secretory IgA, that inhibit microbial survival and growth. Many of these are constitutively expressed but can be upregulated in response to an insult or inflammation. The need for the ocular surface to balance protection and tolerance is highlighted by the fact that the ocular surface contains a unique microbiome with 150 to 200 times fewer bacteria per cell than the adjacent facial skin [4]. The recognition of microbes is mediated by highly conserved pathogen-associated membrane patterns (PAMPs) found on microbes. The ocular surface epithelium detects microbial PAMPs by Toll-like receptors (TLRs) which have been shown to be amplified in DED [5,6,7].

DED is a “multifactorial disease of the ocular surface characterized by a loss of homeostasis of the tear film, and accompanied by ocular symptoms, in which tear film instability and hyperosmolarity, ocular surface inflammation and damage, and neurosensory abnormalities play etiological roles” as defined by the Dry Eye Workshop committee [8]. The committee classifies it into tear deficient and evaporative dry eye. Whether the pathology is caused by deficient tear production and normal evaporation or normal production and increased evaporation, the committee describes the distinction as a continuum rather than a definite separation. Regardless of the classification, hyperosmolarity is a centrally important mechanism. It leads to the activation of signaling pathways such as NF-KB which produce cytokines that further recruit inflammatory cells to release and produce even more mediators [9]. These inflammatory mediators can induce apoptotic death of epithelial cells, loss of goblet cells, and decreased expression of mucins [2]. Loss of glycocalyx mucins can cause early tear break up, further amplifying hyperosmolarity and continuing the “vicious cycle” of DED. Moreover, epithelial damage in this cycle is associated with increased exposure and activation of TLRs, which ultimately produce inflammatory cytokines and subsequently worsen DED findings [5].

DED is marked with a state of chronic ocular surface damage and irritation and activation of the innate immune system. Innate immune activation can have an exacerbating and ameliorating effect on the pathogenesis of DED. Among the salubrious measures initiated by the innate immune system to combat chronic inflammatory damage are measures to improve the healing of epithelium. This is another area where AMPs have been shown to play a significant role in DED. In addition to their roles in the mediation of the inflammatory response, AMPs have been shown to improve wound healing in several models. Epithelial wound healing is an important function of the ocular surface, which is deranged in DED [10]. For example, Histatin-1 improved human corneal limbal epithelial cell migration and wound healing in an in vitro scratch model [11]. Additionally, a functional domain in Histatin-5 was identified that is responsible for the peptides wound-healing properties in a murine corneal wound-healing model [12]. A peptide from a different family of AMPs, Thymosin β4, promoted corneal wound healing after alkali injury in a murine model as well [13]. These experiments mostly followed a similar model to test the peptides efficacy in wound healing. Other groups have studied wound healing at the corneal surface after excimer laser photoablation. Meduri et al. found that basic fibroblast growth factor (bFGF) eye drops significantly increased epithelial healing after excimer photoablation in transgenic mice [14]. Later, utilizing the same model for corneal wound healing, it was found that a topical therapy with bFGF and cytochrome *c* peroxidase (CCP) combination eyedrops was effective in enhancing re-epithelialization after phototherapeutic keratectomy [15]. Both these studies utilized murine models. In a study conducted on human patients after photorefractive keratectomy, another peptide, cysteine, reduced corneal wound-healing time [16]. Mechanistic studies that further explore the wound-healing effects of AMPs at the ocular surface and their interplay with innate immunity in DED are discussed below.

## 2. Antimicrobial Peptides

Human AMPs are small amphipathic peptides ranging from 10 to 149 amino acids in length with an average length of 55 amino acids and an average net charge of +5.6. They mostly possess a positive charge resulting from low acidic, high basic, and hydrophobic amino acid residues. This polarity allows them to disrupt the membranes of various pathogens including Gram positive and negative bacteria, enveloped viruses and fungi [17]. Pore formation in the cell membrane leads to leakage of inner organelle, membrane depolarization, and consequently bacterial cell death. Some AMPs possess killing properties that act intracellularly. This changed the original perception that AMPs needed to be present at high concentrations to effectively execute killing through permeabilization. Presence at lower concentrations was effective because of intracellular actions of the AMPs [18] such as binding specific DNA sequences [19]. Some peptides inhibit DNA and protein synthesis and therefore act as a proteolytic agent inside the bacterial cell [20]. Other peptides act on inhibiting the synthesized proteins.

These peptides are expressed throughout the body including the eyes, skin, mouth, gut, ear, urinary, and nervous systems [17]. AMPs not only respond and protect the ocular surface from environmental pathogens, but they also may possess immune-modulating properties. In addition to their well-cited antimicrobial roles, they are involved in dendritic cell activation [21], complement modulation [22], mast cell histamine release [23], and wound repair [24]. AMPs can stimulate the release of various cytokines such as IFN-α, IFN-γ, IL-2, and IL-13 [25] as well as induce chemotaxis of monocytes [26] and T cells [27] and enhance antibody production [28]. Given their diverse role in the innate immunity of the ocular surface, understanding AMPs’ role in healthy as well as diseased epithelium is of importance to fully comprehending the pathogenesis of OSDs such as DED.

## 3. Distribution/Expression of AMPs in the Ocular Surface

AMPs are well represented at the ocular surface. This presence is either mediated by local epithelial expression, immune infiltration, or as secreted elements in the tear film [29]. Defensins are among the major AMPs present at the ocular surface. They are divided into α-defensins and β-defensins (hBDs). The α-defensins present at the ocular surface are the four related to neutrophils known as human neutrophil peptides (HNPs). HNP-1–3 were first detected in 1998 in inflamed conjunctiva, lacrimal ductules, and tears [30] and their presence was corroborated using liquid chromatography on tear film post ocular surface neoplasm excision [31]. Non-neutrophil α-defensins HD-5 and -6 were absent. As for β-defensins, hBD-1–3 were shown to be expressed by corneal [32] and well as conjunctival [33] epithelial cells. hBD-1 and -3 were expressed constitutively, while hBD-2 was shown to be inducible and only expressed occasionally [32]. Additionally, hBD-2 was recently detected in the tear film [34]. McIntosh et al. were able to detect the presence of hBD-4 in vitro [35] however, this finding was later not corroborated in vivo when utilizing different methods of obtaining samples [36]. Expression of hBD-9 by corneal and conjunctival epithelial cells was interestingly decreased during infection suggesting a non-bactericidal role [37]. Another major AMP with well-cited presence at the ocular surface is LL-37 of the cathelicidin family. It is expressed by both corneal and conjunctival cells [35]. Like HNPs, the level of LL-37 rises in instances of inflammatory infiltration due to its inclusion in neutrophil granules [38]. Along with defensins and cathelicidin, McIntosh at al. reported mRNA expression of LEAP-1 and -2 [35] and Huang et al. found protein level expression of MIP-3a and thymosin beta-4. In addition to antimicrobial species lactoferrin, lysozyme, lipocalin-1, and secretory IgA [39], lacrimal aqueous secretions were found to contain histatin-1 [40]. Ribonuclease-7, an AMP originally found in dermal keratinocytes [41], was expressed in ocular surface epithelial cells at the protein level in infective keratitis samples [42].

## 4. Human Beta Defensins and Cathelicidin

The two major categories of AMPs are defensins and cathelicidins. Human defensins are cysteine-rich peptides of 29–45 amino acids in length held together by sulfide bonds. They are characterized based on the cysteine connectivity pattern into alpha, beta, and theta defensins [43]. The four relevant alpha defensins (human neutrophil peptides 1–4) are produced and stored in azurophil granules in neutrophils where they constitute more than 30% of the protein content [44]. On the other hand, there are 17 known beta defensins, few of which are relevant to ocular surface defense. They show constitutive as well as inducible expression by mucosal and epithelial tissue [43].

Cathelicidins are AMPs that have a cathelin precursor domain resembling the cathelin porcine protein. Porcine cathelin is found in neutrophils and is an inhibitor of the cysteine protease cathepsin L. Human cathelicidin, named LL-37 due to it being 37 amino acids long and beginning with 2 leucine residues, was identified more than two decades ago in cytoplasmic granules of neutrophil leukocytes possessing bactericidal as well as wound-healing properties [45]. Like defensins, LL-37 is present in tissue because of constitutive expression, inducible expression, and immune infiltration [29]. While LL-37 and defensins have been shown to possess broad spectrum microbiocidal properties against bacteria, viruses, fungi, and parasites, for a long time it was thought that issues such as salt sensitivity, concentration, and inactivation by tear film pose physiological threats to their efficacy at the ocular surface [29]. However, multiple studies have challenged that claim and provided evidence of the well-balanced role these peptides play in ocular surface innate defense. In a murine in vivo study, Huang et al. induced *Pseudomonas aeuruginosa* keratitis in cathelicidin knock-out and wild-type mice. The knocked out mice showed significant delayed clearance of the bacteria in the cornea, and an increased amount of infiltrating neutrophils at later times post infection as well as decreased levels of anti-inflammatory interleukins [46]. In another mouse model, silencing of murine beta defensins (human homologs) highlighted the importance of mBD-2 and mBD-3 against ocular *P. aeruginosa* infection [47]. Dua et al. were able to further demonstrate the importance of these AMPs against *P. aeruginosa* and *Staphlococcus aureus* human ocular samples. In response to *P. aeruginosa*, LL-37 and hBD-3 showed an increase expression while hBD-9 showed a marked decrease. However, in response to *S. aureus* multiple tested AMPs showed increased presence but most significantly hBD-2 [48].

Defensins are known to have antiviral activity in various tissues in the body. Alpha defensins depend on their 3D structure held together by disulfide bonds to oligomerize as described previously. On the other hand, beta defensins possess antiviral activity both in their linear as well as 3D form acting in a salt-dependent fashion [49]. Whether these antiviral properties were manifested on the ocular surface was unknown. Abedin et al. were the first to demonstrate a relationship between low levels of hBD-9 and viral keratoconjunctivitis [37]. The same group later tested the expression of several AMPs in conditions of viral keratoconjunctivitis, and they found a marked increase in LL-37 levels along with LEAP-1 mRNA [50].

Antimicrobial peptides are also important for protection against fungal infection at the ocular surface. A study profiling the expression of various AMPs in response to fungal keratitis infection in corneal tissue from patient found that various defensins and cathelicidin were upregulated variably. hBD-1 and -2 had the most consistent expression followed by hBD-3 and -9 and finally LL-37 [51].

## 5. Toll-Like Receptor-Mediated Expression of Defensins

To fully comprehend the role that these AMPs play in the innate immunity of the eye and their importance to OSDs, it is important to study the signaling pathways and mechanistic processes that induce and modulate their expression. TLRs are a family of highly conserved pathogen recognition receptor glycoproteins that are known to recognize PAMPs (e.g., bacterial lipopolysaccharide (LPS), flagellin) on invading microbes [52]. The human genome codes for 10 functional TLRs. All 10 TLRs were found to be expressed on the ocular surface with variable degrees and localizations [53,54]. Expression of AMPs in response to microbial infection is induced by TLR is well documented on the ocular surface. Early studies [55] reported that LPS (TLR4 agonist) from *P. aeruginosa* increases the expression of hBD-2 in human corneal epithelial cells [55]. hBD-2 along with LL-37 showed increased expression in corneal epithelial cells through TLR2 activation of the NF-KB and mitogen-activated protein kinase pathways after exposure to *S. aureus* lipopeptide [56] and heat-inactivated *Fusarium solani* [57]. Activation of the NF-KB pathway by TLR2 ligand on ocular surface epithelial cells was further corroborated [58]. Another AMP induced through TLR2 and the NF-KB and MAPK pathways is hBD-9 [48]. TLR5, known to respond to bacterial flagellin, induced the expression of hBD-2 and LL-37 after exposure to purified *P. aeruginosa* flagellin in corneal epithelial cell lines [59]. Mohammed et al. reported an increase of RNAse-7 expression in response to TLR2, TLR3, and TLR5 activators [49] after their previous studies suggested its involvement in the MAPK and not the NK-FB pathway [42]. Elevated expression of TLR1 through 4 was found in correlation with increased amounts of AMPs S100A8, S100A9, and hBD-1 in epithelial cells infected with *C. pseudodiptheriticum* further suggesting the variability of immune response to different microbes [60]. A later study was conducted using in cathelicidin knock out mice to investigate the role of TLR2 and MyD88 in cathelicidin signaling against *S. aureus* endophthalmitis. TLR2 and MyD88 knock out mice produced lower levels of cathelicidin than the wild type group which left them susceptible to a higher bacterial load for a longer period of time and increased retinal damage. They showed very similar trends of inflammatory mediator expression and bacterial clearance as cathelicidin knockout mice. This led to the conclusion that TLR2 and MyD88 signaling plays an important role in the expression of antimicrobial peptides against bacterial infection [61].

## 6. TLR Expression on the Ocular Surface

**Figure 1:**
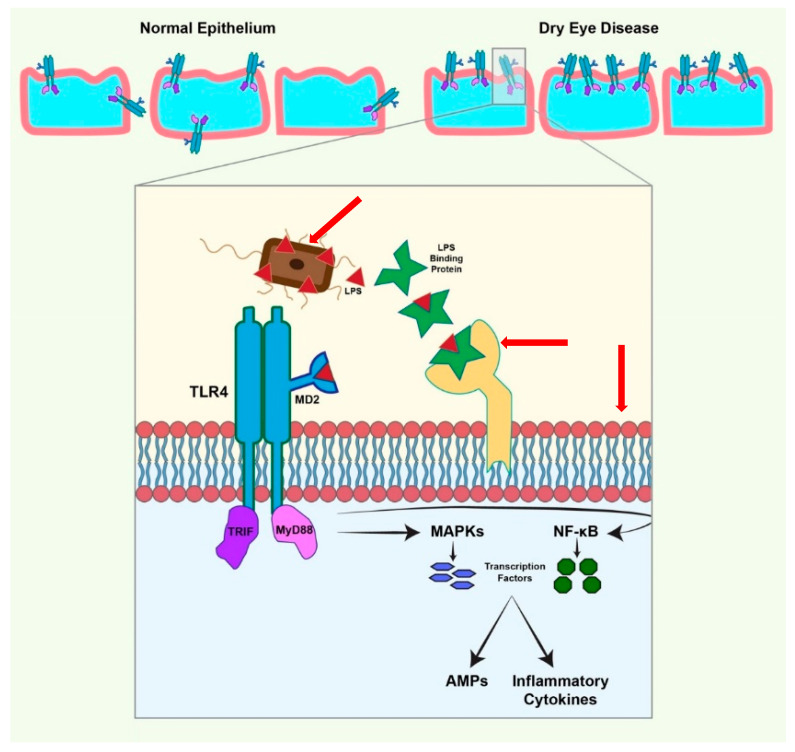
TLR4 Activation is increased in dry eye disease (DED) conditions leading to increased antimicrobial peptides (AMP) and cytokine production in response to bacterial challenge. Disruption of the corneal epithelial barrier in DED leads to the exposure of more TLR4 to the surface leading to greater activation by LPS which leads to an increased production of inflammatory cytokines and AMPs. TLR recognizes PAMPs and induces the production of AMPs (hBD-2 and LL-37) through the MAPK and NF-KB pathways. LPS (red) from bacteria (brown) is recognized by LPS-binding protein which in turn presents it to CD14 (yellow) attached to the phospholipid bilayer. CD14 transfers the LPS to the MD2/TLR4 complex. TLR4 activation is followed by the activation of the NF-KB and MAPK pathway to produce transcription factors that relocate inside the nucleus to induce the transcription of hBD-2 and LL-37.

Published reports have shown somewhat contradicting patterns of TLR expression and that is due to the ocular surface’s ability to stay immune-silent, killing microbes efficiently without excessive inflammation. In 2007, Li et al. showed protein and mRNA levels of TLR-1 through -6 and -9 to be constitutively present in conjunctival, limbal, and corneal epithelial cells while TLR-7, -8, and -10 were absent [54]. However, in 2011 Mohammed et al. reported increased levels of TLR-2 and TLR-8, baseline expression of TLR-1, -3, -5, -6, -7, and -10, and decreased TLR-4 and -9 in bacterial keratitis specimens [50]. Although most of these findings were corroborated by Redfern et al. [53], data on the expression and localization of TLR-2 and -4 were conflicting. Another instance of a similar contradiction in reporting occurred when Li et al. were not able to induce TLR-2 and -4 expression on corneal, conjunctival, or limbal epithelial cells using previously cited PAMPs [54]. TLR-2 responds to peptidoglycan treatment [62], and TLR-4 had shown expression upon corneal exposure to bacterial LPS [63]. As shown in Figure 1, cell surface responsiveness to LPS is mediated by a LPS-binding protein, CD14, and a TLR4-MD-2 complex [64]. An earlier study demonstrated that LPS hypo-responsiveness was the culprit in many conflicting reports because of the lack of MD-2 expression in the corneal epithelium since exogenous MD-2 was capable of restoring the response [65]. For the expression to happen naturally, the availability of MD-2 and LPS-binding protein is mandatory and occurs in response to different microbes. Using MD-2 knockout mice, Roy et al. were able to show that *P. aeruginosa* keratitis induces MD-2 expression and activates LPS responsiveness [66]. The presence of CD14 and soluble LPS binding protein in tear fluid is most likely the last piece of the puzzle [67]. This is specially of importance since it has been shown that TLR4 exposure and activation are increased in DED. The normal proinflammatory response of the epithelium through the activation of TLR4 that leads to the production of AMPs and cytokines was found to be significantly increased in a murine dry eye model. This could be attributed to the increased expression of TLR4 throughout the corneal epithelium, increased expression on the superficial epithelial cells compared to more posterior wing and basal epithelial cells, or perhaps increased exposed of apical TLR from the loss of protective mucins during dry eye disease [5].

## 7. Thymosin Beta

While there exists three families of thymosin polypeptides α, β, and γ, only thymosin β4, β10, and β15 are found in humans. Thymosin β4 is the main peptide found in human tissue and it constitutes 70–80% of the total thymosin content in man [68]. It is a G-actin-binding protein made up of 43 amino acids. It is present in almost all body cells except red blood cells. With an average of 16.3 μg/mL in an extract of whole blood, it is especially of high concentration in white blood cells, platelets, and wound fluid [69]. Tβ4 possess plays an important role in wound healing and in downregulation of inflammation. It promotes repair and regeneration in various tissues including the eye, heart, skin, and central nervous system [70]. It has been shown to decrease cytokines, chemokines, and activation of nuclear factors [71]. Human corneal and conjunctival cells epithelial cells have been shown to express Tβ4 in addition to previously discussed peptides which all contribute to the host defense capabilities of the ocular surface [36].

After Tβ4’s role in dermal wound repair and endothelial cell migration was demonstrated [72], it was hypothesized that it could have similar healing effects at the ocular surface in disease with compromised corneal and conjunctival epithelial integrity such as DED. Experimentation led to the discovery that Tβ4 accelerates in vivo corneal wound healing, modulates corneal cytokine production, and enhances epithelial migration. Rat corneas were debrided with heptanol and after treatment with Tβ4 reepithelization of the wounds and cytokine mRNA transcripts of IL-1β and IL-6 increased showing wound healing as well as anti-inflammatory activity of the peptide [73]. This was then confirmed in an alkali burn model where treatment decreased mRNA transcripts for neutrophils and chemo-attractants MIP-2, KC, and IL-8 and promoted reepithelization [13]. The reepithelization was led by the conjunctival epithelial cell migration promoted through the induction of laminin-5 [71] and TGF-β independently [74]. A recent study testing the effect of Tβ4 on primary human corneal epithelial cells cell migration concluded purinergic signaling involvement. Tβ4 increased HCEC proliferation and migration correlating with increased extracellular ATP levels, intracellular Ca^2+^ influx, and ERK1/2 phosphorylation. This migration was inhibited by P2 × 7 purinergic receptor antagonists. While specific mechanisms are not yet understood, this data guide further studies exploring the role of the purinergic receptor-mediated Ca^2+^ influx in Tβ4-mediated corneal healing [75]. In addition to battling inflammation through the reduction of chemokines, Tβ4 was shown to possess an anti-apoptotic role in epithelial cells by inhibiting the release of mitochondrial cytochrome c which kickstarts apoptosis by activating caspases. This was shown when cells were put under ethanol stress [76] and later benzalkonium chloride stress, a common preservatives in eye drops [77]. However, perhaps the biggest discovery relating to the ant-inflammatory effects of Tβ4 was its impact on the TNF signaling cascade. TNF-α is a proinflammatory cytokine that is upregulated during corneal infection [62]. In a model of TNF-α mediated corneal inflammation, Sosne’s group determined that Tβ4 treatment decreased NF-KB levels and activation. It also reduced p65 subunit phosphorylation as well as blocked nuclear translocation in corneal epithelial cells. While the specific mechanism by which Tβ4 regulates this is poorly understood, it proved very clinically significant since NF-KB is a central mediator of the human innate immune response in all human tissue including the cornea [78]. Once its inactivator Ikβ is phosphorylated, NF-KB is released from its bound-inactive state and translocated to the nucleus to target promoter regions of proinflammatory genes [79]. Qui and colleagues later presented evidence that Tβ4 blocks the NF-KB subunit RelA/p65’s nuclear translocation, targeting the cognate KB side in the proximal region of the IL-8 gene promoter. In addition, it inhibits sensitizing effects of intracellular binding proteins PINCH-1 and ILK on NF-KB activity independent of Tβ4’s actin-binding properties [80]. Together, this evidence drove Tβ4 from mechanistic laboratory studies to the development of therapeutic regimens for the treatment of inflammatory-mediated disorders of the ocular surface. These regenerative properties of the peptide highlight its importance in DED. Using corneal fluorescein staining and sign and symptom assessments, Sosne et al. evaluated the safety and efficacy of Tβ4 eye drops on DED patients in a phase 2 randomized trial. They found statistically significant improvements in signs and symptoms of the patients as well as a reduction in the size of the damaged ocular area. In addition, Tβ4 improved tear film break up time and increased tear volume. These effects were long lasting and propelled the peptide to further trials on larger sample sizes [81]. Additionally, Tβ4 has been shown to be effective in relieving the signs and symptoms of DED in a controlled adverse environment model [82]. Phase 3 clinical trials were announced to commence by the end of 2020 [83]. Jin et al. recently reported the efficacy of glycine Tβ4 eye drops in a murine model of experimental dry eye disease. The eye drop showed significant improvement in all clinical parameters, and there was no significant difference in efficacy when compared to cyclosporine A (CsA)drops [84].

## 8. Histatins

Histatins constitute a family of endogenous AMPs from two genes coding for all extant histatin protein products. They possess antimicrobial, immunomodulatory, and wound-healing properties [85]. It was previously demonstrated that several histatins, most notably histatin-1, are present at higher concentrations than EGF in human saliva and are the primary contributor to the wound-healing effect of human saliva [86]. It has been demonstrated recently that the presence of histatin-1 on the ocular surface could relate to ocular disease states. Histatin-1 was found to be expressed at lower levels in patients with aqueous deficient dry eye disease (Kalmodia et al., 2019). It is thought that histatin-1 is expressed by the epithelium of the accessory lacrimal gland and the main lacrimal gland [87]. Additionally, it has been demonstrated that histatin-1 has a pro-wound-healing effect on human corneal epithelial cells in vitro [11]. These findings were then corroborated in vivo in a rabbit model. Corneal wounds were induced using ethyl alcohol, and the application of topical histatin-1 accelerated the healing process and decreased the wound area compared to controls [88]. Furthermore, histatin-1, -2, and -3 were previously the only histatins known to possess wound-healing capabilities [85]. It was recently discovered that histatin-5 contains a functional domain that is necessary and sufficient to induce cell-migration and promote wound healing both in vitro and in vivo in corneal models [12]. These promising results may suggest a broader role of histatins on the ocular surface and warrant further exploration.

## 9. Lacritin

Lacritin is a human tear glycoprotein that is known to be down regulated in DED [89] and keratitis [90] among other ocular pathologies. It is a 119 amino acid secretory protein that is involved in maintaining cellular homeostasis, cell proliferation, and basal tear secretion. It was first discovered two decades ago after it enhanced exocrine secretion in overnight cultures of lacrimal acinar cells that previously lacked secretory function. Its expression in the eye is limited to the lacrimal functional unit where it is released from lacrimal acinar cell secretory granules [91]. This multifunctional AMP was also found to possess microbicidal capabilities. After challenging human corneal epithelial cells with LPS, Vantaku et al. found that supplementation with exogenous lacritin halted LPS-induced cell death through the mediation of COX-2 [92]. In addition to its cytoprotective capabilities, this AMP is able to maintain homeostasis at the ocular surface. This is achieved through its ability to halt autophagy, relief epithelial stress, and resume metabolism. FOXO3 is a transcription factor involved in regulating apoptosis and under stress and initiates apoptosis after translocating to the nucleus. A recent study found that cultured corneal epithelial cells treated with dry eye basal tears (which are void of lacritin) stimulated FOXO3 translocation to the nucleus. Addition of lacritin was able to relocate the apoptotic transcription factor back to the cytoplasm highlighting lacritin’s importance in maintaining homeostasis at the ocular surface [93]. In addition, lacritin possesses proliferative and wound-healing properties. It was found to be mitogenic in vitro on nongerminal epithelia derived from tissue locations that regularly encounter lacritin [94]. Another study reported enhancement of cellular uptake, calcium-mediated signaling, and closure of scratch in HCE cells treated with lacritin in vitro. They confirmed these findings in vivo and showed that topical administration of a lacritin-based peptide promoted corneal wound healing and epithelial integrity in non-obese diabetic mice [95]. For lacritin to perform its cytoprotective roles, binding to ocular surface epithelial cell surface protein syndecan-1 is necessary [96]. In normal tear fluid, lacritin monomers, dimers, trimers, and other multimers have been detected [97]. These multimers result from tissue transglutaminase cross-linking where glutamine 106 is an acceptor. Given that glutamine 106 is part of the syndecan-1 binding domain on the peptide, multimers are not able to efficiently bind syndecan-1 and have decreased or show nonexistent bioactivity [98]. Therefore, the presence of lacritin monomer is essential for the peptide to perform its functions. In dry eye disease, tissue transglutaminase expression is markedly increased [99]. Since tissue transglutaminase is responsible for the cross-linking multimers, its increased expression should decrease lacritin monomer levels in dry eye disease [100]. Lacritin was found to be deficient in aqueous deficient dry eye (ADDE) [89] and Sjögren’s syndrome (SS) DED [101]. The correlation between decreased lacritin tear levels and SS has been reported to be of high diagnostic sensitivity and specificity for the syndrome [102]. Vijmasi et al. reported that application of topical lacritin promoted tear secretion, reduced infiltration of lymphocytes, and restores ocular surface integrity in Aire-deficient mice. Their study provided the first in vivo data to support the use of lacritin in cases of dry eye [103]. Another study used CsA to induce endogenous expression of lacritin in rats. It was shown that 0.05% CsA induced lacritin expression and lead to the improvement of signs and symptoms of dry eye in a chronic pseudo-immune rat model of dry eye [104]. Recently, Georgiev et al. reported that C-terminal forms of lacritin are deficient in dry eye and are essential in preventing tear film collapse and instability. Immuno-depletion of C-terminal proteoforms from normal tears led to the loss of tear stability. Application of C-terminal as opposed to N-terminal proteoforms in a rabbit model restored stability to these tears as well as dry eye tears. These findings may be a result of the potential role of this portion of lacritin in preventing film collapse and restoring epithelial homeostasis [105]. In addition, a double masked, randomized clinical trial was initiated in 2017 to test the viability of a synthetic peptide based on lacritin’s C-terminal in cases of primary SS DED. The clinical trial is still ongoing [106].

## 10. Role of AMPs in Dry Eye Diseases

DED is a complex group of diseases that have a central feature of a defective tear film and deranged ocular surface homeostasis associated with epithelial and immune dysfunction. Hyperosmolarity of the tears appears to be an important driver of inflammation, which can lead to epithelial cell dysfunction and death, reduction of goblet cell numbers, and decreased expression of mucins [2]. Although an area of debate, the normal tear film is represented by a simple three component model. The first component is mucin which reduces the hydrophobicity of the ocular epithelium. The mucins that are in contact with the epithelium prohibit attachment of debris and pathogens and stabilize the tear film while maintaining hydration. This mucin layer is formed of membrane-associated mucins and gel-forming mucins. MUC1 [107], MUC4 [108], and MUC16 [109] make up the membrane-associated mucins and are produced by the cornea and the conjunctiva. Gel-forming MUC5AC and MUC19 are produced by goblet cells [110]. In an in vitro model of corneal epithelial cells, Menon et al. demonstrated that reduced expression of membrane associated mucins MUC1 and MUC16 showed marked increase production of cytokines and inflammatory mediators in the presence of TLR agonists and bacteria flagellin [111]. This suggests that these mucins contribute to the maintenance of innate immunity homeostasis through the suppression of TLRs at the ocular surface. As previously discussed, TLR activation is responsible for the intracellular pathways that lead to the expression of AMPs. Therefore, modulating the presence of mucins should inversely affect the expression of AMPs on the ocular surface through the suppression or activation of TLRs.

Various studies have demonstrated the effect of DED on ocular surface mucins (Table 1). First, the loss of goblet cells decreases the production of gel-forming mucins. A decreased concentration of MUC5AC has been shown in DED tears [112] as well as decreased conjunctival expression [113]. In addition, expression of membrane-associated mucins MUC1, MUC4, and MUC16 were shown to be significantly decreased in DED [31,113,114]. Given their role in the suppression of TLRs, a decrease in mucins in DED should lead to an increase in the expression of various AMPs due to the inhibition of the suppression of TLRs. However, the exact pattern of mucin expression in DED is not fully understood. In SS, which can be characterized by severe dry eye, a different pattern of expression is found. Gel-forming mucins act similar to non-SS DED. Argüeso et al. reported decreased levels of MUC5AC mRNA in conjunctival epithelial cells as well as reduced levels in tear fluid [115] in SS patients. Similarly, gel-forming MUC19 was also decreased in expression and production [110] in SS patients. However, membrane associated mucins showed a different trend. Increased levels of MUC1 [116] and MUC16 [117] were found at the mRNA and the protein level in patients with SS when compared with patients with other forms of dry eye and controls. This can perhaps be attributed to the difference of severity of dry eye that causes an increase in expression as a defense mechanism.

**Figure 2:**
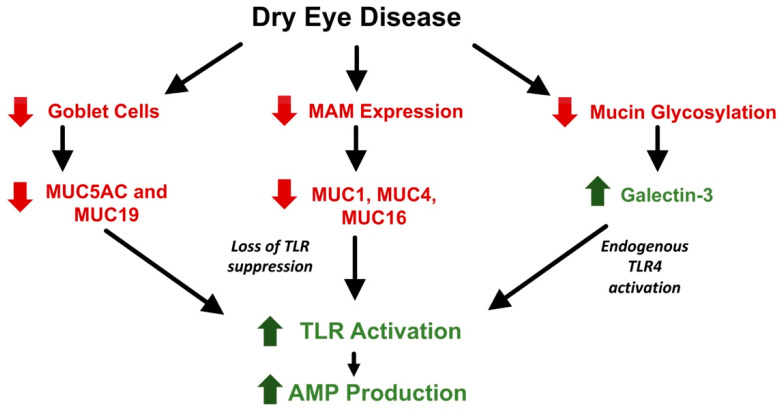
Effect of dry eye disease on AMP production through TLR modulation. DED causes a decrease (red arrow) in goblet cells that produce gel-forming mucins MUC5AC and MUC19. There is also a decrease in the expression of membrane-associated mucins MUC1, MUC4, and MUC16. Since mucins suppress TLR activation, their decrease may lead to an increase in TLR activation. In addition, impaired mucin glycosylation during DED causes the release/increase (green arrow) in the level of the galectin-3 which acts as an endogenous activator of TLR4. This overall increase in TLR activation leads to an increase in AMP production.

In healthy ocular surface epithelium, membrane-associated mucins perform their protective and structural functions through their interaction with galectin-3. This carbohydrate-binding protein binds MUC1 and MUC16 on the apical surface of epithelial cells. Decreased levels of galectin-3 are associated with the loss of the epithelial barrier function and the increase in permeability [118]. Uchino et al. found an increase in galectin-3 levels in tears associated with epithelial dysfunction in DED patients. Expression of the protein was unchanged, however [119]. The authors attributed this to the alteration of mucin glycosylation in DED leading to the release of the bound galectin-3. Galectin-3 is recognized as a pattern-recognition receptor (PRR) as well as a danger-associated molecular pattern (DAMP). PRRs and DAMPs play crucial roles in the innate and adaptive defense-activating TLRs as well as recruiting immune cells [120]. Galectin-3 was shown to be an endogenous TLR4 ligand activating the receptor without the need for exogenous factors such as LPS [121]. Therefore, in DED, there is a decrease in mucin expression as well as an increase in galectin-3 release into the tear film which may lead to the activation of TLRs and their associated pathways. This could theoretically lead to an increase in AMP expression in DED (Figure 2) as studies have shown subjects with moderate DED [122] and SS DED [123] showed increased gene expression of hBD-2. The exact stimuli on the ocular surface that upregulates AMP expression during DED remains unclear, but it also likely to attributed to cytokines as well as PAMPs or DAMPs in the tear film. 

A study analyzing tear fluid of DED subjects using iTRAQ quantitative proteomics found that AMPs of the S100 calcium-binding family, S100 A8 and S100 A9 to be significantly increased in the tears of DED subjects [124]. The authors also found S100 A8 and S100 A9 were useful to differentiate the severity of dry eye (based on tear breakup time times and maybe a potential biomarker for DED [124]. Interestingly, a recent report demonstrated significantly decreased levels of histatin-1 in tears of ADDE patients. The report suggested that the strength of the correlation could allow the use of histatin-1 as a biomarker for ADDE [40]. Additionally, hBD-9 showed a slight decrease in expression in non-SS DED [37]. This further proves the association between AMPs levels and DED. While the exact pattern of change is not fully understood, the reduction in protective mucin may allow for additional TLR activation and production of AMP when the ocular surface is compromised.

Thus, there are many studies indicating differential expression of various mucins and AMPs in DEDs. This differential expression is sometimes elevated, as might be expected in response to environmental pathogens, sometimes reduced, and sometimes associated with different forms of AMPs becoming predominant in disease states. Increases or decreases in protein and mucin content could reflect different stages or severities of disease or be associated with genetic or environmental factors that are yet to be elucidated. AMP production changes patients with DED appear to be specific to each different protein or mucin and reflect the complex roles these proteins play in ocular surface homeostasis.

## 11. AMPs in Other OSDs and Therapeutic Avenues

AMPs in the ocular surface are also relevant to other OSDs and could represent potential therapeutics of multiple OSDs. One example is the use of thymosin beta 4 in cases of neurotrophic keratopathy. Neurotrophic keratopathy is a degenerative disease marked by decreased corneal sensitivity, altered tear composition, impaired corneal wound healing, ulceration, and perforation. After several successful preclinical studies [125], a formulation of Tβ4 was moved up to clinical trials. In 2020, RegenerX reported results of a phase 3 (SEER-1) clinical trial showing significant improvement in NK after utilization of the drug.

A recent study reported the efficacy of the use of an AMP loaded ocular insert for infectious keratitis. Although their synthetic peptide derived from lactoferrin was unstable, the freeze-dried matrix they developed for the inserts showed promising potential as a good delivery vehicle for AMP treatments in the precorneal area [126].

In addition, AMPs have a potential for use in contact lens coating and solutions. Dutta et al. demonstrated that melamine-coated contact lenses reduced the incidence of *P. aueriginosa* microbial keratitis on contaminated contact lenses [127]. The same group conducted another experiment bonding LL-37, Mel-4, lactoferrin, and melamine to poly-hydroxyethylmethacrylate (HEMA) lenses. They found that none of the peptides proved to be cytotoxic to the murine ocular cells, and the peptides significantly reduced the growth of *S. aureus* and *P. aeruoginosa* [128].

## 12. Conclusions

AMPs protect the ocular surface from infection when the ocular surface is compromised from desiccation or during infection to maintain homeostasis. Endogenous AMPs are an important aspect of the ocular surface, with roles to play in microbial defense, innate immune modulation and response, and epithelial homeostasis. Basic science investigations into understanding the mechanisms of action of AMPs in DED and ocular surface homeostasis have begun to yield significant translational importance, with multiple AMPs being tested in clinically relevant models of disease and in clinical trials. While there is still much that is not understood about the specific mechanisms and pathways that AMPs are involved in on the ocular surface, there has been significant progress in translating and expanding existing knowledge from the bench to the bedside. Further study of AMPs as important contributors to ocular surface physiology and as potential new treatments are necessary.

## Figures and Tables

**Table 1:** Modulation of mucins and AMPs in dry eye disease.

*Molecule*	Non-Sjögren DED	Sjögren DED	References
**Gel-forming mucins**			
**MUC5AC**	Decrease	Decrease	[113,115]
**MUC19**	-	Decrease	[110]
**Membrane associated mucins**			
**MUC1**	Decrease	Increase	[113,116]
**MUC4**	Decrease	-	[113]
**MUC16**	Decrease	Increase	[114,117]
**Galectin-3**	Increase	Increase	[119]
**Antimicrobial Peptides and Proteins**			
**hBD-2**	Increase	Increase	[122,123]
**hBD-9**	Decrease	-	[37]
**Histatin-1**	Decrease	Decrease	[40]
**Lacritin**	Decrease	Decrease	[89,101]
**S100 A8**	Increase		[124]
**S100 A9**	Increase		[124]
